# Reducing Amputations in People with Diabetes (RAPID): Evaluation of a New Care Pathway

**DOI:** 10.3390/ijerph15050999

**Published:** 2018-05-16

**Authors:** Sandra MacRury, Kate Stephen, Fiona Main, Jane Gorman, Sandra Jones, David Macfarlane

**Affiliations:** 1 Institute of Health Research and Innovation, University of the Highlands and Islands, Inverness IV2 3JH, UK; kate.stephen@uhi.ac.uk; 2 Highland Health Board, Inverness IV2 3UJ, UK; fiona.main2@nhs.net (F.M.); jane.gorman2@nhs.net (J.G.); sandra.jones7@nhs.net (S.J.); d.macfarlane1@nhs.net (D.M.)

**Keywords:** diabetes, Technology-Enabled Care, prevention, foot care

## Abstract

People with diabetes are at increased risk of foot ulcers, which, if left untreated, can lead to infection, gangrene, and subsequent amputation. Management by a multidisciplinary diabetes foot team has been shown to reduce amputation rates; however, accessing specialist treatment is made particularly difficult when living in remote and rural locations, such as many individuals cared for within NHS Highland. The RAPID project was made up of two phases: firstly, to evaluate the technical feasibility of a new integrated care pathway using innovative technology, and secondly, to establish process enhancement of the pathway to justify a larger-scale study. Omni-Hub^TM^ enabled a face-to-face consultation by the community podiatrist to be enhanced by virtual consultation with members of the multidisciplinary foot team, including specialist diabetes podiatrists and a diabetes consultant. The technical feasibility study provided recommended changes focused around adaptations to the equipment used and the best means to gain successful connectivity. The process enhancement study demonstrated positive outcomes in the process with positive effects both in the service received by patients and experiences of healthcare professionals involved. The RAPID project provides evidence and justification for a larger-scale empirical study to test an embedded pathway and technology solution, which will inform policy change and a paradigm shift in the management of foot ulceration in the community.

## 1. Introduction

Diabetes foot disease is a major cause of morbidity and mortality [1]. People with diabetes have a high incidence of foot ulceration related to neuropathy and ischemia, which has led to an amputation rate of 40% [2]. Foot ulcers in diabetes are a preventable condition, yet they precede more that 80% of amputations [3]. Foot ulcers and amputations are the leading cause of diabetes-related hospital admissions estimated at 47% [4], with an alarming proportion of 50% of those who have had a major amputation shown to die within two years following their procedure [5].

It is estimated that £60 million is spent on foot ulcers and amputations in Scotland alone [6], highlighting the critical importance in preventative and early intervention strategies to improve foot care in this population.

NHS Highland covers the largest and most sparsely populated areas of the UK, representing 42% of the landmass in Scotland. The service faces unique challenges due to the rural and remote location of many of its residents, who are faced with limited or no access to the services they require due to the distances to/from health services and transport limitations. Connectivity issues across the region further compound the issue, meaning that already existing technology-enabled care (TEC), such as web-based self-management programmes and/or interventions, can be difficult to access due to poor web connectivity where patients reside.

Early intervention strategies have been shown to be cost effective in the treatment of diabetes foot disease [7], as has the role of introducing multidisciplinary teams in the reduction of amputation rates [8]. There is evidence that an integrated foot care pathway, with trained staff in foot protection services in the community and speedy access to multidisciplinary specialist teams, considerably lowers the risk of amputations [6].

Accessing such strategies in NHS Highland can be difficult due to a large proportion of patients covered by the health board living in rural and remote locations that can hinder accessibility to specialist clinics and teams. Widening access to multidisciplinary teams through innovative solutions to reduce travel and provide specialist care within the community could contribute to improved care and reductions in amputations rates. Furthermore, developing such a care pathway aligns with the Diabetes UK (DUK) campaign stressing the importance of ‘putting feet first’ in diabetes care [9] and Scotland’s 2020 vision to enable everyone to live longer, healthier lives at home, or in a homely setting, ensuring that wherever that setting is, care is provided to the highest standard of quality and safety [10].

This evaluative pilot project aimed to meet this need by developing and testing an innovative integrated care pathway using enhanced technology to improve connectivity and enable remote access to specialist care. The project was broken down into two phases: an initial technical feasibility study and small-scale pilot study (RAPID-1), and an interim study to provide process enhancement and detailed evaluation (RAPID-2). The aims of RAPID-1 were to establish a care pathway system that prompted community podiatry staff or community nurses to use the specific email link created to seek advice from the diabetes specialist foot team regarding management of patients with a diabetes foot ulcer under their care. This allowed early triage and, where necessary, remote review of people with diabetes-related foot problems by a multidisciplinary team through innovative use of technology in a remote and rural health setting. Remote review was via a video consultation link to the patient’s home facilitated by a network router stored in the community podiatrist’s car outside the patient’s home. The aims of RAPID-2 were to provide process enhancement to permit a larger-scale study of an embedded pathway and technology solution (integration planning) which will inform policy change and a paradigm shift in the management of foot ulceration in the community.

## 2. Materials and Methods 

**Figure 1:**
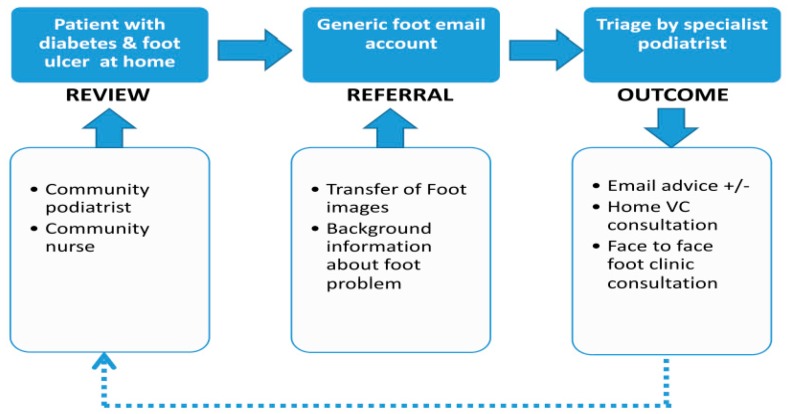
RAPID pathway: the process flow for community patient referral and triage to specialist diabetes foot services.

**Figure 2:**
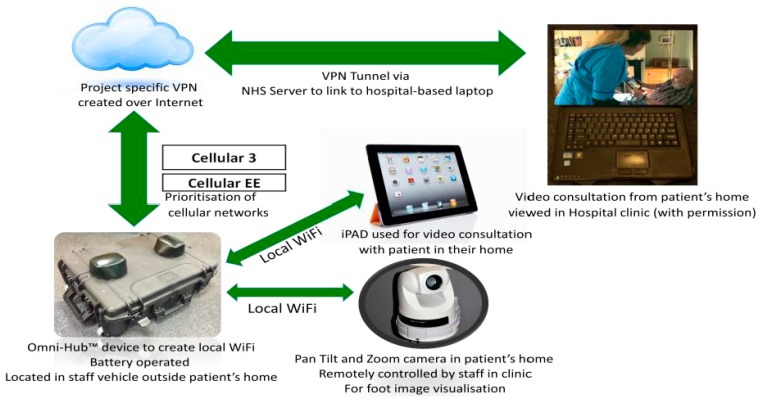
The technology configuration used in the RAPID-1 pilot project.

The RAPID project team consisted of two community podiatrists, responsible for community consultations and collection of patient questionnaire responses; three diabetes specialist podiatrists, responsible for foot image collation, email triage, and data collection related to specialist consultations, ulcer grading, and outcomes; two diabetes consultants based within the Highland Diabetes Institute. The process is demonstrated in Figure 1. A rural health post-doctoral researcher was responsible for staff interviews, collation, and analysis of data. An initial training session was held to provide practical demonstration, supplemented by operating instructions, guidance notes, and follow-up sessions between our industry partner, Tactical Wireless (TWL), the project coordinator, and the community podiatrists. Please see Figure 2 for the technical configuration of the RAPID pilot project. The focal equipment, provided by TWL, used in the project was the Omni-Hub^TM^ integrated communication system, a portable unit capable of optimizing priorities for WiFi, multiple cellular networks, or satellite. The project received ethical approval from the University of the Highlands and Islands ethics committee, number ETH830.

### 2.1. RAPID-1: Technical Feasibility Pilot

The initial technical feasibility pilot was conducted for 3 months between January and April 2016. In this phase, the Omni-Hub^TM^ was initially positioned on a TWL-owned and -driven vehicle combined with an Axis cube camera. As the study progressed, the equipment was adapted to allow each community podiatrist to access a separate Omni-Hub^TM^ and a pan-tilt-zoom (PTZ) camera that could be controlled from the specialist team at the Highland Diabetes Institute (HDI).

Eligible participants were individuals with active foot ulceration residing in the localities covered by the study, recruited sequentially as part of their normal care by the community podiatrists or nurses in the area who were then referred to RAPID to receive the enhanced care pathway. Patient information was accessed and shared through the diabetes database SCI-Diabetes [11].

Feasibility and acceptability were assessed across four areas: (i) overall practitioner experience using a data-capturing pro forma; (ii) individual patient experience using a short questionnaire administered by the community podiatrist or telephone survey after the consultation; (iii) technology-specific usability through discussions with the specialist foot team; and (iv) triangulation of data from the aforementioned stages with technical quality and clinical data on treatment pathway and impact of the intervention.

### 2.2. RAPID-2: Process Enhancement Pilot

Following recommendations from the initial technical feasibility study, the process enhancement pilot was conducted for 5 months from November 2016 to March 2017.

The equipment consisted of two Omni-Hub^TM^ integrated communication systems to allow WiFi boosting used in conjunction with an NHS tablet installed with appropriate applications to connect with the Omni-Hub^TM^ to control the camera(s) and enable video conferencing (VC). The equipment was used to send information, including still or video images, to the specialist team for review and triage, or to connect live with the diabetes clinic as required. The Omni-Hub^TM^ also allowed for the use of voice over Internet protocol (VOIP) with smartphones enabling telephone calls.

Eligible participants were patients with diabetes and their carers with an active diabetes-related foot ulcer, residing in areas with poor connectivity issues (highlighted in RAPID-1).

The project was evaluated in relation to the stated aim of process enhancement, which involved gathering data across four areas: (i) the treatment of ulcers; (ii) the use of technology in relation to connectivity, quality, and utility; (iii) external or other factors that impacted the process; and (iv) qualitative data on the attitudes and experiences of stakeholders.

The treatment of ulcers was assessed using a data-capturing pro forma at each visit which included details of the foot ulcer, antibiotic treatment, and wound management. The form was saved as a PDF and community podiatrists used an app on the tablet to complete and submit the form. Use of Community Health Index (CHI) numbers on this form allowed for linkage with historic patient data.

The use of technology, as above, was reported using a data-capturing pro forma for each visit by the community podiatrist. The form included sections on aspects of the process of using the technology to capture and send images and for live links with the specialist team. Data was also collected around factors that may have contributed to the quality of the connections, with the option to include additional data around the specific causes of delays and/or any problems encountered.

Qualitative data was collected via telephone structured interviews from a template using categorised responses per question, to explore attitude and experiences of stakeholders who had interacted with the project; these included general community podiatrists and community nurses in the locations who had referred patients into the process, the specialist diabetes podiatrists, and physicians in the multidisciplinary teams. Interview questions, devised and agreed by the project team, were around the practical usability of the technology, access and connectivity, acceptability of support at both technical and specialist clinical levels, and time resource. For the community-based staff who engaged with patients using the new service, questions were around the process of referring, the outcome for the patient, and views about the likelihood of referring patients to a potential future service or similar services for other conditions.

Data gathered from the aforementioned methods were then analysed and triangulated using a realist approach with quantitative data matched with qualitative data to establish what worked and in what conditions. Given the community-based nature of the use of technology, nonclinical factors which impacted on the service delivery and process were also reported.

## 3. Results

### 3.1. RAPID-1: Technical Feasibility Pilot

Thirty-two patients were referred to the RAPID-1 project, resulting in 95 patient contacts by the community podiatrists. Of these 95 contacts, a live link to the diabetes clinic at HDI was attempted 15 times. Of the remaining 80 contacts, the equipment was used to send information and images to the specialist team in the clinic at HDI. The mean age of the patients was 65 years, and the range was 47 to 86 years. Nine participants were female and 24 were male. All patients had type 2 diabetes. One patient had an ischaemic ulcer, with 16 demonstrating neuro-ischaemic ulcers, and 14 neuropathic ulcers, with missing data for one patient. Patients lived between approximately 13 and 106 miles from the HDI, with an average return trip of 90 miles.

There were initial difficulties in establishing successful live links which were overcome through a period of testing and support between TWL and the RAPID team. Collaboration between TWL and the RAPID team was successful in iterative development and problem solving throughout the study and this was identified as a crucial aspect for inclusion in the RAPID-2 pilot.

Findings from the technical feasibility study were recorded across three areas: equipment use, impact on work load, and impact on patients and patient care.

### 3.2. Equipment Use

**Figure 3:**
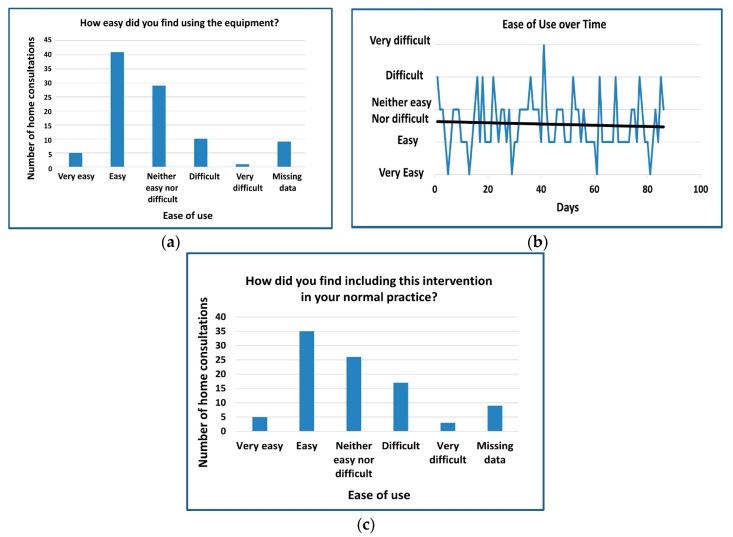
Graphs demonstrating ease of use of RAPID equipment for each home consultation: (**a**) community podiatrists’ responses as to whether they found the equipment easy to use at each home consultation; (**b**) changes in ease of use as the study progressed; (**c**) responses from community podiatrists for each home consultation to ease of including the intervention into normal practice.

Overall the RAPID team reported that that they did not find the equipment difficult to use, with a trend line towards increased use over the time within the feasibility study (Figure 3). However, there were some technical issues highlighted that allowed for recommendations and adjustments to be made for the follow-on process enhancement pilot. These initial technical difficulties were discouraging; with delays in connection and communication challenges both within and without the RAPID team. Although aspects of the project were challenging at times, confidence grew as the study progressed and live links were more successful.

Challenges were seen mainly in communicative and administrative processes rather than the effectiveness of the equipment itself. The team saw “massive potential” in the technology and expressed appreciation for their ability to participate in the project. It was also discussed that carrying lots of equipment, setting up, and using unfamiliar technology posed further challenges with suggestions given to have lighter and less pieces of equipment in the follow-on study.

The use of the Omni-Hub^TM^ located within the podiatrist’s car created connectivity difficulties during some visits, necessitating repeated trips between the car and the patients’ homes to reposition the vehicle. Lastly, it was noted that there were difficulties in operating the camera in terms of needing to reposition it, and repeatedly changing gloves and rewashing hands.

### 3.3. Impact on Workload

The main impact upon workload was noted in time, with the importance of live links fitting within normal consultation times stressed as a means to ensure maximum benefit. Feedback forms were not completed for all 95 repeat visits from the patient home visits due to a combination of time constraints and failed connectivity. Eighty out of 86 feedback forms from the community podiatrists reported an increased consultation time with a relatively even split between ‘little time’ (*n* = 42) and ‘a lot of time’ (*n* = 38). Despite these responses in the majority of patient contacts, the community podiatrists did not report it to be difficult to include in their normal practice.

### 3.4. Impact on Patients and Patient Care

The most prominent success of the pilot was seen in the impact on patients and the care they received, through quicker diagnosis, improved accuracy of diagnoses, and improved access to treatment. Community staff recorded patients’ responses to the intervention after each consultation. Overall, 31 were very accepting, 43 accepting, and 12 neutral.

The RAPID team clearly identified a benefit to patients in not having to travel to the hospital with at least 2892 miles of patient travel avoided, and specialist advice received far earlier as a result through involvement in the RAPID project.

Benefits were also seen through earlier intervention of pressure-relieving aids and earlier referral to orthotics. The potential to refer to a vascular consultant earlier and for weekly live consultations with the diabetologist was reported to have worked well within the project. The system of triage developed for RAPID-1 was successful, with improved follow-up of the database, such as checking microbiology results and antibiotic safety net system.

Unexpected additional benefits were seen from the increased contact between community podiatrists and the specialist diabetes team at HDI specifically around the strength of antibiotics prescribed. Specialists identified that some prescriptions were too low to be effective and the dose was increased appropriately. Similarly, a further unforeseen benefit observed was that patients were more likely to receive off-loading devices much earlier on in their treatment pathways, which was identified as having “massive potential” in improving patient care.

Lastly, the potential for the service to be used in the prevention of foot ulcers in high-risk patients in addition to triage and treatment of patients with active foot ulceration was discussed, and one patient would have been admitted to hospital had they not received treatment through RAPID.

### 3.5. RAPID-2: Process Enhancement

Following the changes identified in the technical feasibility study, a further process enhancement study was conducted with 26 patients over a total of 65 patient contacts. The patients’ ages ranged from 47 to 89 years, and the mean age was 60 years. All patients had type 2 diabetes. Ten participants were female and 16 were male. Some of the patients developed a foot ulcer during the project period; two were triaged by the project within a week of developing their ulcer. The duration of ulcers ranged from 1–4 weeks (*n* = 9), 1–3 months (*n* = 3), 3–6 months (*n* = 5), to over 6 months (*n* = 7).

### 3.6. Patients, Community Podiatry, and Integrated Care Teams

Five patients were seen only once, four were seen twice, eleven had three visits, two had five visits, and one had six. One patient died soon after referral before being seen, and information from two patients was unavailable. Of the outcomes reported, there was a significant improvement in six patients, one ulcer healed with vascular intervention, and one healed without.

There were high levels of satisfaction reported from staff involved in the project, with particular emphasis on the inability for many people to travel the significant distance to be seen by the specialist teams at HDI with the “massive difference” the pilot project made, especially for high risk and very remote patients discussed. It was noted that although community nursing teams hold expertise, this input is generic and “the specialist input was absolutely of benefit to the patient”. The same community nurse expressed her hope for the project to be extended, as “it’s been tremendous for the patient and all the team involved”.

During this project period, one patient was admitted to hospital for a minor amputation, however, it should be noted that this patient had had previous amputations, with multiple wounds. It was projected that without the involvement of the RAPID team, this would likely have been a major amputation, since the admission was on the recommendation of the diabetes consultant based on the live link with the patient.

The timeliness of decision-making around hospital admissions was highlighted as a benefit of RAPID. These hospital admissions, in the same light as the minor amputation, should not be viewed in a negative light. Instead, they were a necessary intervention to avoid deterioration of the wound. Without the timeliness of the intervention facilitated by the RAPID project, it is likely that the wounds would not have healed as well or as quickly, although clearly the project was not designed to assess this.

Further to the physical benefits to patients, there were positive impacts upon mental health and self-management discussed. The social isolation of many patients was particularly noted, with the home visits presenting an opportunity for interaction which patients readily embraced. Staff involved in the project described that “some patients were depressed” and “in a rut” with poor self-care but that the RAPID “visits were positive and encouraged them to self-manage”.

In addition to the benefit to patients, the community-based staff reported a positive impact on their work, which highlighted the process enhancement from the RAPID-1 project. Staff described the project as taking “the pressure off”, and another described the benefits of the immediacy with which contact to the specialist team could be made, and that it was “good to be able to access the specialists. You definitely feel more supported in decision-making. You know they are there, normally, but this helped in an instant way”. This was reiterated by another community podiatrist who described the specialist input as “reassuring” and giving “confidence that I was following the right advice, doing the right thing”.

### 3.7. Technology and Connectivity

Improvements to the technology and refining of the process as learned from the RAPID-1 project were recognised as making things “easier” and “more straight-forward”. The use of tablets to take photos required fewer glove changes and a lighter, more portable Omni-Hub^TM^ improved the functionality of the project as a whole. Although challenges were still encountered with technology and connectivity, this was vastly improved, with 43 of the 65 contacts reporting no problem or delay in connectivity, 44 reporting no problems identified with the tablet, and 46 reporting no relevant or highlighted connectivity issues. The few technical issues raised during this pilot were around the positioning and movement of the tablets cameras and the Omni-Hub^TM^.

Still images were taken using the tablet to be sent to the RAPID triage team by HDI as well as the NHS Medical Image Management System (MIMS). Podiatrists reported that “there was amazing sharpness of image” when using the tablet. In total, 177 images were taken, of which 116 were then selected to send to the HDI and MIMS. It was noted that lighting conditions varied and podiatrists used their initiative to modify lighting to ensure sufficient quality of images. Despite the overall ease of use and positive feedback regarding the use of the tablets, it was noted that there were still challenges around the physicality of working in this way when alone in a domestic environment, with one podiatrist stating that “taking an image could be tricky if the patient was in bed, especially taking an image of an ulcer on the heel whilst trying to lift the leg”.

Live links were tested using different VC systems throughout the project to maximize learning; these were Jabber (embedded in NHS Highland), VSee, and Crossfire. When connectivity was good and all three were tested, all were successful. However, VSee was considered to be “the best” as it “worked better and was more straight-forward” and performed the best when connectivity was established but connections were weak.

Connectivity was seen to be the main challenge in the successful establishment of live links, this was an improvement from RAPID-1, which identified challenges in *using* the technology. The positioning of the Omni-Hub^TM^ was found to be crucial in connectivity, with requirements to move the hub from the podiatrist’s car to within the domicile, or vice versa, and parking availability demonstrating challenges, although the “slimmed down, simplified” Omni-Hub^TM^ made the process of establishing a connection far easier. Nonetheless, in some cases there was a need to ask patients to move within their home, but in some cases patients were bed-bound or in a nursing home so this was not always possible. Lastly, in addition to the position of the hubs and patients, the locations of the patients’ homes or the community health centre were found to impact the success of a connection, with some pockets of NHS Highland identified as geographical areas where even Omni-Hub^TM^ was unable to reach. It was noted that mapping of these areas would be useful to allow options for alternative or additional connectivity to be investigated.

When it was not possible to achieve a live link, telephones were used to facilitate consultation through staff–staff and staff–patient communication. Although this ensured consultation still went ahead, it was noted that VC “was better than just speaking on the phone about the photo. The face-to-face was better for the consultation”. The process for the live links was improved from the previous RAPID-1 project, ensuring consideration of time in terms of durations and scheduling of consultations, keeping consultation times within 20 minutes with successful connection and scheduling live links at the end of the clinic to provide more flexibility with connectivity. The clinic side of the process consisted of the following steps:

1. Image emailed to RAPID team
2. Connection established—image examined
3. When connected, opened up consultation and spoke to patient
4. Advice given
5. End of live link

### 3.8. Further and Future Rollout

Feedback about potential further and future rollout was unanimously positive, with rollout very much welcomed, recognising the benefits for “very remote, rural area(s)” with “patients who are not accessing the services they need” as a result. It was widely recognised that the use of technology would “benefit their patients” and that this was welcomed “as long as the service was secure, then absolutely”.

Further to the improvement in patient care, it was highlighted that the use of technology within the community “would help make community healthcare more efficient”. It was noted that any initial “cost of the investment would soon be covered by savings of this efficiency”, although it was clear that appropriate “training in the use of equipment [should be] given”. The notion of using the RAPID technology to facilitate a mini, mobile office to enable out-of-office administration related to home visits was identified as a huge benefit. This was especially true for peripatetic staff that either did not have a base or where their base was some distance from the patients’ homes. A ‘portable office’ was identified as improving access to relevant patient information which would improve treatment and administrative efficiency, although it was also noted that doing such administrative tasks from the patient’s home was not an option as it would be an intrusive extension to the duration of a home visit.

## 4. Discussion

In this pilot project, an initial technical feasibility pilot (RAPID-1) was successful in establishing a care pathway using innovative technology to allow for early triage and, where necessary, remote review and treatment of people with diabetes-specific foot problems with a specialist multidisciplinary team. The RAPID-1 pilot identified challenges in using this technology to improve foot care in people with diabetes, providing considerations and recommendations for a further process enhancement pilot (RAPID-2). The RAPID-2 project demonstrated positive effects in the use and delivery of the adapted and improved technology-enhanced service, successfully providing a process enhancement and justification for a larger-scale testing of an embedded pathway and technology solution (integrated planning) which will inform policy change and a paradigm shift in the management of foot ulceration in the community.

This preliminary evaluative project is the first project of its kind testing the use of the Omni-Hub^TM^ to enhance diabetes foot care in rural and remote locations within NHS Highland. Although this is a key strength to the study, in the same light, the very nature of the project being in its infancy means that the study findings are mostly anecdotal rather than empirical. However, the methods used in this evaluative pilot project were effective in achieving the stated aims of identifying the technical feasibility of the care pathway and influencing a process enhancement around technology-enabled, community-based management of diabetic foot ulcers in rural and remote locations, warranting further investigation using more rigorous testing methods.

A further strength of the RAPID care pathway was its use of technology in a way that does not place the burden of technological engagement on the patient. TEC interventions are often focused around self-management and/or self-monitoring interventions using online portals, programmes, and/or devices [12]. While such TEC strategies can be hugely effective in improving health, they can be equally exclusionary for individuals with little experience or confidence in using such means for engagement. As demonstrated in the current findings, a large proportion of individuals requiring such innovative care are in the older generations, which is further compounded by their rural and remote locations. It has been shown that barriers to technology are more prevalent in older individuals due to decreased motivation, and lack of confidence and/or skills [13], as such; the RAPID project demonstrates an encouraging means to improve care for such individuals in an inclusionary and progressive way.

The need for specialist multidisciplinary input in improving diabetes foot care outcomes has been widely recognised in national guidelines [3] and recommendations from empirical evidence [6,7,8]. The RAPID project has demonstrated an innovative means to allow patients living in rural and remote locations, with limited to no access to specialist input, a way to engage with specialist multidisciplinary teams to allow for triage, specialist advice, and early intervention.

The improvements seen in the RAPID project show great potential in improving quality of life for patients living with diabetes. Foot ulcers and amputations are highly impacting to an individual’s experience, with detriment seen to self-esteem, quality of life, and increased depression [14]. This improvement can be seen, not only through improved ulceration outcomes, but also in interactions with staff and reduced isolation. In a survey conducted on behalf of Diabetes UK, it was found that more than half of people living with diabetes did not realise that having a foot ulcer put them at more risk of having an amputation [9]. The increased interaction with specialist staff for people living in rural and remote locations through the RAPID project has the potential to improve knowledge and understanding and subsequent self-management.

The monitoring mechanisms from the RAPID project were designed to capture problems and delays in process, implementation, and in connectivity. While this was the main focus of the findings, the benefit to patients should not be underestimated and further work is needed to test the refined care pathway in a way that analyses the impact on patient outcomes more thoroughly. The RAPID project makes ten recommendations for the testing of the pathway further in potential future research:

1. Further refinement of technology to facilitate optimum connectivity is required.
2. On-site support from IT experts for, or prior to, initial home visits may alleviate pressure on health professionals in testing optimum factors to establish a connection. This could include identification of the best locations for the Omni-Hub™ and the patient within the home, local health centre, or clinic.
3. Mapping connectivity at the homes of high risk patients likely to require a domestic consultation and information on patients’ records about connectivity from their homes would help to streamline methods of establishing a connection and to identify patients for whom a domestic live link consultation was not possible.
4. Opportunities for rollout to other professionals within integrated care teams would be useful to increase cost-effectiveness of the technology, increase benefit to rural patients, and to identify and train willing staff in each locality.
5. Eligibility criteria for home visits by community podiatrists and others within integrated care teams should be reviewed to manage variation. The case for home visits for individuals with diabetic foot ulcers should be made clear.
6. Training in the use of the technology on-site and easily accessible, specialist Information, Communication and Technology support would increase capacity and confidence in staff.
7. Increased awareness of and training around wound management of diabetic foot ulcers is required to avoid inappropriate type and frequency of dressing changes and to reduce unnecessary home visits. Increased awareness should help to increase referrals to Diabetes Specialist Podiatrist to allow triage, specialist input, and timely treatment.
8. Unscheduled admissions to hospital of patients with diabetic foot ulcers and amputations should not be categorised as entirely negative as there are occasions when these are timely interventions which avoid further deterioration. Evaluation of the RAPID project and other community-based interventions should be seen in this light.
9. A database of ‘high risk’ patients should be maintained and used to manage proactive, preventative interventions.
10. Protocols for home visits and live links and pathways for knowledge transfer of expert advice in the treatment of diabetic foot ulcers should be refined and disseminated.

## 5. Conclusions 

The RAPID project has successfully developed and tested the technical feasibility and enhanced the process of an innovative technology-enabled care pathway to improve care in people with diabetes and foot ulcers. Although the study is a preliminary pilot project with methodological limitations, it has demonstrated improved patient and staff experience, with better access to specialist advice and consultation, and early intervention and treatment for people living in remote and rural locations who would otherwise have difficulty accessing such care. It lays important and compelling foundations for a larger-scale study to test an embedded pathway using innovative technology underpinned by a health economic assessment which could inform policy change and a paradigm shift in the management of foot ulceration in the community in keeping with the Scottish Government’s 2020 Vision of people being cared for in their home or homely setting.
